# IGF1R promotes radiation-induced HSCs activation by regulating DNA-PKcs-mediated DNA damage repair

**DOI:** 10.3389/fcell.2025.1678654

**Published:** 2025-11-14

**Authors:** Jiguo Lin, Gang Zhao, Jie Feng, Chaonan Sun, Chang Liu, Jiajing Li, Yannan Shen, Yunyun Cheng

**Affiliations:** NHC Key Laboratory of Radiobiology, College of Public Health, Jilin University, Changchun, China

**Keywords:** insulin-like growth factor 1 receptor, DNA damage repair, DNA-dependent protein kinase catalytic subunit, hepatic stellate cells, ionizing radiation

## Abstract

**Introduction:**

Ionizing radiation (IR)-induced liver fibrosis is one of the most serious complications of radiotherapy for liver cancer, and the core of its development lies in the activation of hepatic stellate cells (HSCs). The insulin-like growth factor 1 receptor (IGF1R) is commonly known as a growth-promoting kinase receptor that plays a critical role in cell differentiation and tissue reorganization, as well as in promoting the activation of HSCs, tentatively. Additionally, there has been a resurgence of interest in its role in DNA damage repair; nevertheless, the underlying mechanism remains poorly understood. Considering that DNA damage and repair are the most serious radiation injury events, the aim of this study was to explore the mechanism of IGF1R in the activation of HSCs by regulating DNA damage repair.

**Method and results:**

In this study, we first confirmed that IR induced the activation of HSCs, along with DNA damage and the upregulation of DNAdependent protein kinase catalytic subunit (DNA-PKcs) and IGF1R expressions. Then we indicated that the radiation-induced activation of HSCs and DNA damage repair were promoted by the activation or overexpression of IGF1R, either alone or together with DNA-PKcs activation, mechanistically through IGF1R–DNAPKcs interactions. The process is primarily facilitated by the nuclear translocation of IGF1R, which promotes *PRKDC* transcription at the mRNA level. Moreover, it involves an interaction with DNA-PKcs in the cytoplasm at the protein level, which, in turn, facilitates the entry of DNA-PKcs into the nucleus and subsequent promotion of DNA damage repair.

**Discussion:**

Our findings suggest that the inhibition of the IGF1R-promoted, DNA-PKcs-dependent non-homologous end joining (NHEJ) repair mode is a promising strategy to prevent the activation of HSCs. To the best of our knowledge, the present study is pioneering in its exploration of the mechanism by which IGF1R mediates radiation-induced activation of HSCs by regulating DNA-PKcs.

## 1 Introduction

Ionizing radiation (IR)-induced liver fibrosis represents a critical pathological manifestation of radiation injury, characterized by intricate molecular signaling networks and cellular interactions, with hepatic stellate cells (HSCs) activation playing a pivotal role (Higashi et al., 2017; Mederacke et al., 2013; Coll et al., 2018; Aydın and Akçalı, 2018; Niu et al., 2023). The activation of HSCs is a dynamic, multistage process driven by diverse molecular and cellular interactions, broadly divided into initiation and perpetuation phases (Horn and Tacke, 2024). During the initiation phase, HSCs activation is triggered by inflammatory cytokines and lipid peroxidation products released from injured liver cells (Matsuda and Seki, 2020; Gao et al., 2022). These factors enhance the sensitivity of HSCs to fibrogenic stimuli, while oxidative stress and extracellular matrix (ECM) remodeling, such as basement membrane disruption, induce transcriptional reprogramming, endowing HSCs with proliferative and migratory capabilities (Fan et al., 2019; Geng et al., 2023). In the perpetuation phase, autocrine and paracrine feedback loops sustain activation. Activated HSCs secrete pro-fibrotic mediators, which reinforce their activation to promote collagen-I and collagen-III synthesis and suppress ECM degradation by downregulating matrix metalloproteinases (MMPs) (Fan et al., 2019). Simultaneously, PDGF signaling via the PI3K/AKT and MAPK pathways drives HSCs proliferation and migration, while NF-κB-mediated inflammatory responses amplify the pro-fibrotic phenotype and stabilize the transition from quiescence to activation (Wang et al., 2023; Tao et al., 2022; Cheng et al., 2020).

Morphologically, activated HSCs undergo a striking transformation, losing vitamin A-storing lipid droplets (Li et al., 2025), expanding their rough endoplasmic reticulum and Golgi apparatus (Bai et al., 2024), and adopting a myofibroblast-like morphology characterized by smooth muscle actin (α-SMA) expression (Hon et al., 2013). Functionally, they shift from producing basement membrane collagens (types IV and VI) to secreting fibrillar collagens (types I and III), leading to pathological ECM deposition. This imbalance is exacerbated by their secretion of tissue inhibitors of metalloproteinases (TIMPs), which suppress MMP activity, further impairing ECM degradation (Benyon and Arthur, 2001). Notably, early intervention targeting injury-induced inflammatory signals or oxidative stress during the initiation phase could disrupt the fibrogenic cascade before irreversible ECM remodeling occurs, offering a promising therapeutic avenue for reversing hepatic fibrosis.

Liver fibrosis, a pathological process marked by ECM deposition following chronic liver injury, is closely associated with persistent DNA damage and impaired repair mechanisms (Liu et al., 2025; Zhou et al., 2023). DNA double-strand breaks (DSBs), the most severe form of DNA lesions, are predominantly repaired via non-homologous end joining (NHEJ) in mature hepatocytes (Chang et al., 2017; Yang et al., 2023). The DNA-dependent protein kinase catalytic subunit (DNA-PKcs), a core regulator of NHEJ, not only safeguards genomic stability but also plays context-dependent roles in liver fibrosis. Recent studies have revealed that DNA-PKcs leads to mitochondrial DNA damage, reactive oxygen species (ROS) generation, and subsequent HSCs activation and ECM deposition (Zhou et al., 2019). Conversely, aberrant DNA-PKcs activity, whether through hyperactivation or functional deficiency, may induce error-prone NHEJ repair, resulting in genomic instability and aberrant amplification of pro-fibrotic genes (Zhao et al., 2025; Mao et al., 2023). Intriguingly, efficient NHEJ-mediated DSB repair can mitigate DNA damage accumulation in HSCs, thereby suppressing fibrosis signaling and delaying fibrosis progression (Chang et al., 2017). Given the predominance of NHEJ in HSCs and the dual role of DNA-PKcs in liver fibrosis, elucidating whether its pro-survival or pro-fibrotic function dominates in radiation-induced DNA damage holds profound implications for understanding the molecular drivers of the activation of HSCs.

The insulin-like growth factor 1 receptor (IGF1R), a transmembrane tyrosine kinase receptor, comprises two subunits (extracellular ligand-binding domains) and two ß-subunits (intracellular kinase domains), exhibiting high homology with the insulin receptor (Riedemann and Macaulay, 2006; Scharf and Braulke, 2003). Its ligands include IGF1, IGF2, and insulin, with IGF1 demonstrating the highest binding affinity (Chrudinová et al., 2024; Wu et al., 2022). Current therapeutic strategies targeting the IGF1/IGF1R axis primarily rely on competitive receptor blockade, exemplified by monoclonal antibodies such as teprotumumab and small-molecule inhibitors such as IGF1C peptide, which have shown efficacy in oncology and cardiovascular diseases (Wei et al., 2024). Notably, recent evidence highlights the potential of IGF1R inhibitors in mitigating liver fibrosis (Jiang et al., 2023; Stauffer et al., 2024). Furthermore, TGF-β/Smad signaling downstream of IGF1R exacerbates fibrotic ECM deposition (Wang et al., 2023; Sun et al., 2024). Intriguingly, IGF1R, traditionally recognized as a membrane-bound signaling receptor, has been observed to translocate to the nucleus (Poreba and Durzynska, 2020), although its nuclear functions remain enigmatic. Additionally, IGF1R physically interacts with DNA-PKcs, a core regulator of NHEJ repair (Yang et al., 2020; Yang C. et al., 2021), yet whether this interaction modulates DNA damage response remains unexplored. Critically, the role of IGF1R in radiation-induced DNA damage repair and its potential crosstalk with the activation of HSCs remain elusive.

Given the pro-fibrotic roles of IGF1R in both fibrogenic signaling and potential regulation of DNA repair, elucidating its precise mechanisms in post-radiation DNA damage resolution through *in vitro* experiments holds transformative potential for understanding hepatic fibrogenesis. This knowledge could unveil novel therapeutic targets to disrupt the vicious cycle of DNA damage, activation of HSCs, and ECM remodeling.

## 2 Materials and methods

### 2.1 Cell culture and ionizing radiation

The human hepatic stellate cell line (Lieming Xu-2, LX-2) and the rat hepatic stellate cell line (HSC-T6) were purchased from BioChannel Biotechnology Co., Ltd (China). The LX-2 cells and HSC-T6 cells were cultured in RPMI 1640 medium (Gibco, United States) and Dulbecco’s modified Eagle’s medium (DMEM), with high glucose separately containing 10% fetal bovine serum (FBS, Gibco, United States) and 2% penicillin/streptomycin (p/s, Solarbio, China) at 37 °C in a humidified atmosphere of 5% CO_2_. The culture medium was supplemented with the desired drugs: IGF1R agonist at 10 ng/mL (IGF1, MCE, United States), IGF1R inhibitor at 10 μM (AZD-3463, KKL, China), and DNA-PKcs inhibitor (STL127705, MCE, United States) as needed. Subsequently, 2.22 Gy/min of X-ray radiation was administered to cells using PXI X-RAD (United States).

### 2.2 Immunofluorescence

Cells were cultured on Lab-Tek culture slides in six-well plates in a complete medium. After irradiation of adherent cells, the slides were removed and permeabilized with 4% paraformaldehyde and 0.5% Triton X-100 for 15 min and blocked in 5% BSA for 1 h at 37 °C. The slides were then incubated with the following primary antibodies overnight at 4 °C: anti-53BP1 antibody (1: 200, CST, United States), anti-γ-H2AX antibody (1: 200, CST, United States), anti-IGF1R antibody (1: 200, CST, United States), and anti-DNA-PKcs antibody (1: 200, CST, United States). Subsequently, cells were incubated with the FITC-conjugated secondary antibody (1: 200, CST, United States) and the rhodamine-conjugated secondary antibody (1: 200, CST, United States) for 1 h at 37 °C. The cell nucleus was counterstained with DAPI (Servicebio, China) for 20 min, and images were captured using a laser scanning confocal microscope (Nikon, Japan). Finally, fluorescence intensity was quantified using ImageJ software.

### 2.3 Alkaline comet assay

The alkaline comet assay was performed to quantify the severity of DNA damage. Three hours after irradiation, the cells were harvested and resuspended in phosphate-buffered saline (PBS) and then mixed with low-melting-point agarose gel at 37 °C. Then, the gel containing the embedded cells was meticulously layered over a microscopy slide that had been previously coated with 1% normal-melting-point agarose gel. The cells were lysed in lysis solution (comprising lysis buffer and DMSO at a 9:1 ratio) overnight at 4 °C. The slides were immersed in alkaline electrophoresis buffer for 60 min at room temperature, and then, alkaline electrophoresis was performed in alkaline electrophoresis buffer at 25 V for 30 min. The slides were neutralized three times with neutral buffer at 4 °C and then stained with 20 μl of propidium iodide solution for 30 min in the dark. Finally, comet images were captured using a laser scanning confocal microscope (Nikon, Japan).

### 2.4 Wound healing assay

The wound healing assay was performed to compare cell migration abilities, which reflect the activation of HSCs. In brief, the cells were seeded in a six-well plate at a density that allowed them to reach 80%–90% confluence within 24 h (approximate 2–5 × 10^5^ cells/well). After irradiation treatment, a straight scratch was made across the well using a 10 µL pipette tip held vertically and gently dragged through the cell monolayer immediately. Subsequently, the cells were washed with PBS, and serum-free medium was used to replace the complete medium to minimize the effect of cell proliferation. Finally, the images were captured at regular intervals (0 h, 24 h, and 48 h) at the same location using an optical microscope. The migration areas were measured using ImageJ software.

### 2.5 Boron difluoride dipyrromethane staining

The loss of intracellular lipid droplets indirectly reflects the activation level of HSCs. The lipid droplets in irradiated HSCs were detected using boron difluoride dipyrromethane (BODIPY) staining. The steps were as follows: cells were seeded in a six-well plate at a density of 10%–20% confluence within 24 h before irradiation. The BODIPY working solution, sufficient to cover the cells, was added and incubated at 37 °C in the dark for 30 min. Subsequently, the staining solution was removed and replaced with fresh medium, followed by observation under a fluorescence microscope (Nikon, Japan).

### 2.6 Phalloidin staining

Phalloidin staining specifically binds to F-actin in the cytoskeleton, thus labeling cytoskeleton distribution and morphology, which can be used to observe cytoskeleton dynamics during cell morphology changes, migration, division, and adhesion. The cells were cultured on Lab-Tek culture slides in six-well plates in a complete medium. After irradiation of adherent cells, the slides were removed and permeabilized with 4% paraformaldehyde and 0.5% Triton X-100 for 15 min and blocked in 5% BSA for 1 h at 37 °C. Then, the slides were incubated with FITC-Phalloidin (MCE, United States) overnight at 4 °C. The cell nucleus was counterstained with DAPI (Servicebio, China) for 20 min, and images were captured using a fluorescence microscope (Nikon, Japan).

### 2.7 Dual-luciferase reporter assay

The dual-luciferase reporter assay is a widely used technique for gene expression regulation studies. It is mainly used to detect the role of promoter/enhancer activity in the regulation of gene transcription and can verify whether a specific transcription factor binds to the target DNA sequence and regulates gene expression. The PGL3-basic reporter vector containing the promoter of DNA-PKcs and the internal reference plasmid pRL-TK were co-transfected into LX-2 cells seeded in 24-well plates at a ratio of 10:1. The assay was carried out 48 h after transfection. After discarding the medium, cells were lysed by adding 100 µL of lysis buffer per well, and the supernatant was taken after centrifugation at 15,000 rpm. A volume of 100 μL of the supernatant was taken in a 96-well plate, mixed with Dual-Lumi Firefly Luciferase Detection Reagent, and incubated at room temperature for 5 min to detect the fluorescence signal. Subsequently, 100 μL of Dual-Lumi Renilla Luciferase Assay working solution was added to the same reaction tube to quench the firefly signal and activate Renilla luciferase, and the fluorescence signal was immediately detected using Cytation 3 (BioTek, America). All steps were performed according to the instructions of the Dual-Lumi™ Luciferase Assay Kit (Beyotime, China). Finally, statistical analysis was performed by calculating the ratio of firefly luciferase activity to Renilla luciferase activity.

### 2.8 Construction of IGF1R overexpression- and knockdown-stable HSCs

Lentivirus vectors containing IGF1R coding sequence and IGF1R-interfering fragments, purchased from Public Protein/Plasmid Library (China), were used to construct stable HSCs with IGF1R overexpression or IGF1R knockdown, designated OE or sh-IGF1R, respectively. The lentivirus vectors were transfected twice, 24 h apart, when the cells reached 60% confluence. Then, the cells were cultured in virus-containing medium with puromycin for 2 weeks, with the medium changed every 2 days. The efficiency of IGF1R overexpression and knockdown was verified by Western blot.

### 2.9 Quantitative PCR analysis

After functionally graded radiation, cells were lysed using TRIzol reagent (Invitrogen, United States). The total RNA was extracted using chloroform and isopropanol and dissolved in RNase-free water to a final volume of 20 μL. The concentration of RNA was determined using a microplate reader (BioTek, United States) with absorbance wavelength at 260 nm. The reverse transcriptional reaction was performed using an Evo M-MLV RT Mix Kit (Takara, Japan) with gDNA clean, and the real-time quantitative PCR process was performed according to the manufacturer’s instructions. The following primer sequences were used in this experiment: for IGF1R (human) (forward: TCGCACCAATGCTTCAGTTC; reverse: GGAGGGTTCCACTTCACGAT) and for PRKDC (human) (forward: CAGAAGATCGCACCTTACTCTGT; reverse: ACTTAATAAGAAGGTCCAGG GCT).

### 2.10 Nuclear–cytoplasmic fractionation

According to the manufacturer’s protocol of the Nucleoprotein Extraction Kit (BBI, China), the cells were treated with a hypotonic buffer. Cytoplasmic protein lysate is obtained by centrifugation of the supernatant. The precipitate remaining after centrifugation was treated with lysis buffer to obtain nucleoprotein lysate.

### 2.11 CO-IP

The Protein A/G Magnetic Beads (MCE, United States) were used to determine the binding of IGF1R with DNA-PKcs. The magnetic beads were pre-incubated with 400 µL of IGF1R antibody(CST, United States) for 24 h in advance. After the irradiated cells were lysed using 600 µL of lysis buffer, 200 µL was taken as the input group, and the remaining lysate was co-incubated with pre-incubated magnetic beads overnight. On the following day, the magnetic beads were collected, to which 50 µL 1×SDS-PAGE loading buffer was added, and the supernatant was taken as the experimental group. The binding protein level was detected using the Western blot assay.

### 2.12 Western blot analysis

All the irradiated cell samples were extracted using cell lysis buffer containing 1% protease inhibitors and phosphatase inhibitors to obtain total protein. To evaluate the levels of differentially expressed proteins, 8% sodium dodecyl sulfate–polyacrylamide gel electrophoresis (SDS-PAGE) was used to separate extracted total proteins. The blocked PVDF membrane, which transferred total proteins, was incubated overnight at 4 °C with primary antibodies specific to IGF1R (CST, United States), DNA-PKcs (CST, United States), α-SMA (Proteintech, China), collagen-1 (Proteintech, China), XRCC4 (Proteintech, China), XLF (Proteintech, China), GAPDH (Proteintech, China), and HRP-conjugated secondary antibody (BBI, China) for 1 h at room temperature. The aimed protein signals were detected and captured using the Mini Chemiluminescent System (SINSAGE, China) with a high-sensitivity ECL Kit (Beyotime, China). Quantitative analysis of grayscale was performed using GenoSens Analysis (Clinx Science Instruments, China), and GAPDH was used for normalization.

### 2.13 Statistical analysis

All statistical analyses were performed using GraphPad Prism 8.0 software (San Diego, United States), and the data were presented as mean ± standard error. A *p*-value <0.05 was considered statistically significant ( * indicates *p* < 0.05, ** indicates *p* < 0.01, and *** indicates *p* < 0.001). All experiments were performed and repeated independently at least three times.

## 3 Results

### 3.1 Determination of IR-induced HSCs activation

**FIGURE 1:**
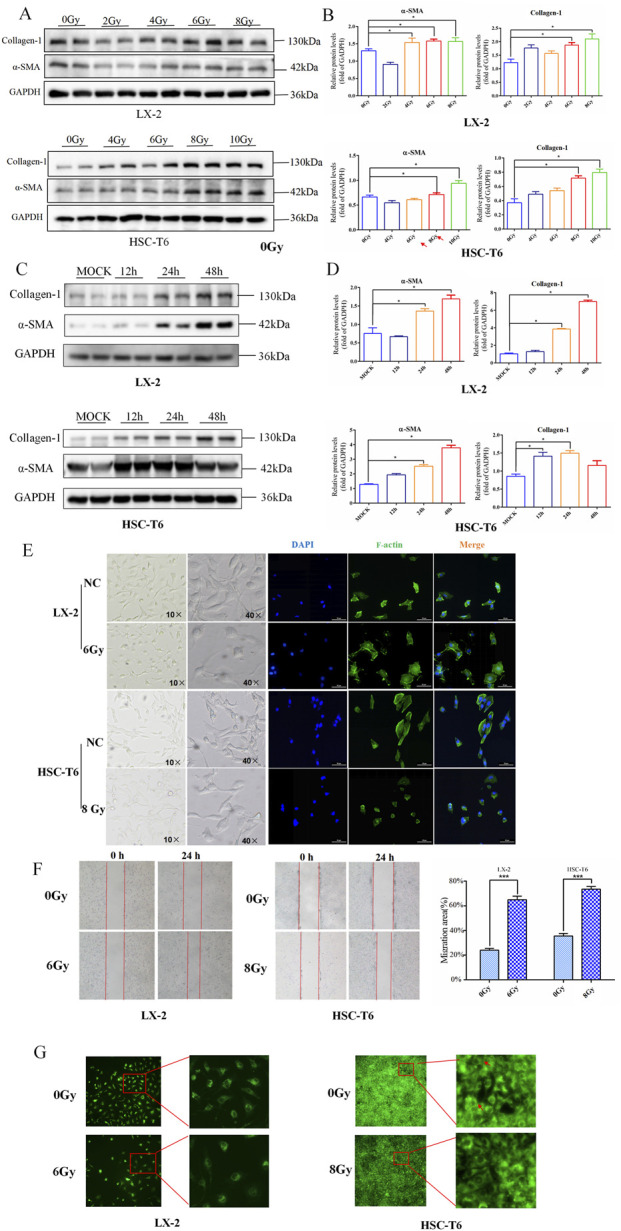
Determination of IR-induced HSCs activation. **(A)** Western blot measured the level of collagen-1 and α-SMA in LX-2 and HSC-T6 cells after dose-gradient treatment with IR. **(B)** The quantitative analysis of the collagen-1 and α-SMA levels in LX-2 and HSC-T6 cells after dose-gradient treatment with IR. **(C)** Western blot measured the levels of collagen-1 and α-SMA in LX-2 and HSC-T6 cells at a time-gradient following IR treatment. **(D)** The quantitative analysis of the levels of collagen-1 and α-SMA in LX-2 and HSC-T6 cells at a time-gradient following IR treatment. **(E)** The cell morphology in the bright-field state of the microscope and cytoskeleton staining by IF; the cell nuclei were counterstained with DAPI (blue), and the cell cytoskeleton was stained with phalloidin (green). **(F)** The migration capability of LX-2 and HSC-T6 cells after IR treatment, measured using the wound healing assay. **(G)** The intracellular lipid droplet content of LX-2 and HSC-T6 cells after IR treatment, measured using BODIPY staining.

We first measured the classical markers of HSCs activation in gradient-dose IR to investigate the optimal dose of HSCs activation in HSC-T6 and LX-2 cell lines. As shown in Figures 1A,B, upregulation of α-SMA and collagen-1 indicated significant activation of HSC-T6 and LX2 in response to higher doses of radiation. Hence, regarding the almost comparable expression pattern of these two different indicators under the gradient doses, we ultimately determined 6 Gy and 8 Gy as the optimal doses for irradiation on LX-2 and HSC-T6 cells, respectively.

It is well known that the temporal dynamic effects of IR inevitably lead to the differential expression of factors associated with altered cellular phenotypes in a time-dependent manner. To further verify whether temporal dynamics contribute to differences in the timing of HSCs activation, we detected these markers in a time gradient after irradiation. The results of Western blot revealed that the onset of α-SMA and collagen-1 often perform over a longer period of time (Figures 1C,D). Therefore, 24 h after irradiation was chosen as the most significant time point for IR-induced HSCs activation-related phenotypes in HSC-T6 and LX-2 cells.

Then, to further confirm the dose and time suitable to activate HSCs, functional assessments were performed to detect four hallmark alterations associated with myofibroblast transdifferentiation following irradiation exposure to LX-2 and HSC-T6 cells. The results showed that exposure of HSCs to irradiation led to an enhanced collective migration capacity (Figure 1F) and marked depletion of intracellular lipid droplets (Figure 1G). In addition, the cytoskeleton underwent reorganization after irradiation, but the morphological changes manifested differently in HSC-T6 and LX-2 cells. In the HSC-T6 cell line, this was characterized by cytoskeletal disruption and a substantial reduction in focal adhesions, whereas in the irradiated LX-2 cell line, the number of synapses decreased, and the cell body increased (Figure 1E).

Collectively, these findings established the optimal experimental paradigm for modeling radiation-associated HSCs activation dynamics in LX-2 and HSC-T6 cell lines.

### 3.2 DNA damage involved in irradiation-induced HSCs activation

**FIGURE 2:**
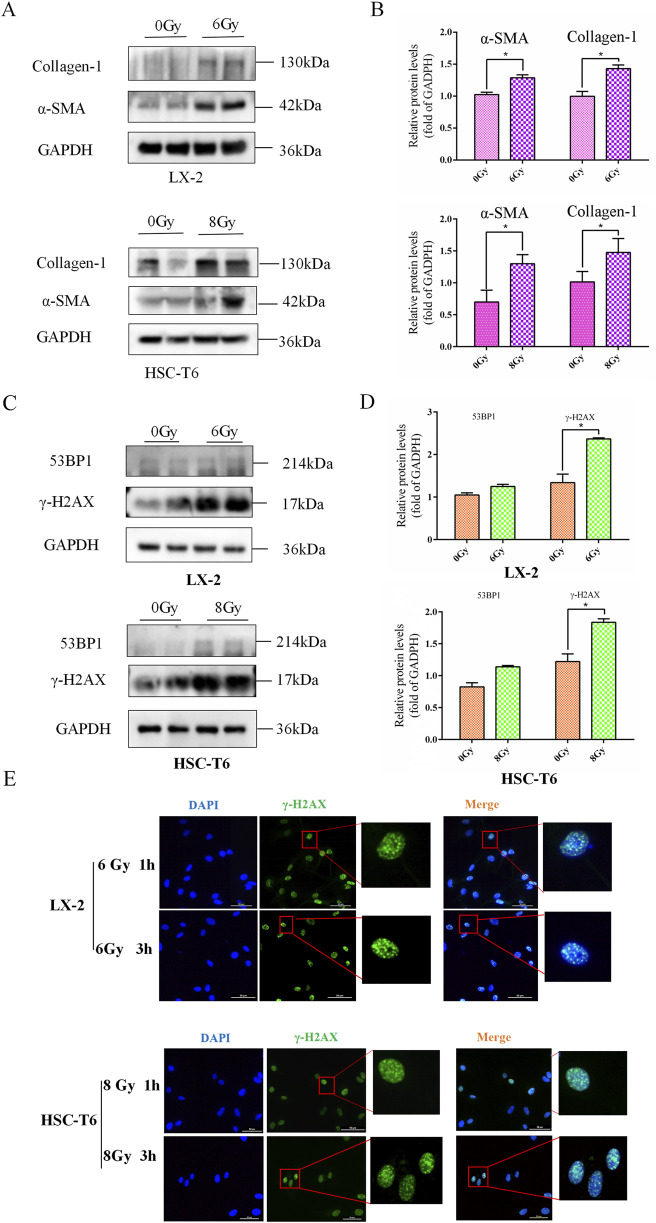
DNA damage involved in irradiation-induced HSCs activation. **(A)** The levels of collagen-1 and α-SMA in LX-2 and HSC-T6 cells after 6 Gy or 8 Gy irradiation, measured using Western blot. **(B)** The quantitative analysis of the levels of collagen-1 and α-SMA in LX-2 and HSC-T6 cells after 6 Gy or 8 Gy irradiation. **(C)** Western blot measured the levels of 53BP1 and γ-H2AX in LX-2 and HSC-T6 cells after 6 Gy or 8 Gy irradiation. **(D)** The quantitative analysis of the levels of 53BP1 and γ-H2AX in LX-2 and HSC-T6 cells after 6 Gy or 8 Gy irradiation. **(E)** DNA damage foci of LX-2 and HSC-T6 cells were detected with IF of γ-H2AX 1 h or 3 h after irradiation.

As persistent DNA damage lesions are recognized drivers of radiation-induced liver pathology, it is imperative to elucidate whether irradiation triggers stress-associated DNA damage checkpoint activation in HSCs since such radiobiological events may establish a mechanistic link between DNA damage and HSCs activation. Following the establishment of a significant correlation between IR and HSCs activation, the subsequent investigation focused on ascertaining whether this manipulation resulted in HSC damage and DNA damage in the early phase following IR treatment. Subsequently, the results of the Western blot revealed that IR induced high-levels of 53BP1 and γ-H2AX expressions (Figures 2A–D). Meanwhile, the immunofluorescence (IF) results of γ-H2AX foci further confirmed that DNA damage exists in the process of HSCs activation after IR treatment (Figure 2E), indicating a potential regulatory role for DNA damage in response to HSCs activation.

### 3.3 IR led to the upregulation of IGF1R, and IGF1R promoted IR-induced HSCs activation

**FIGURE 3:**
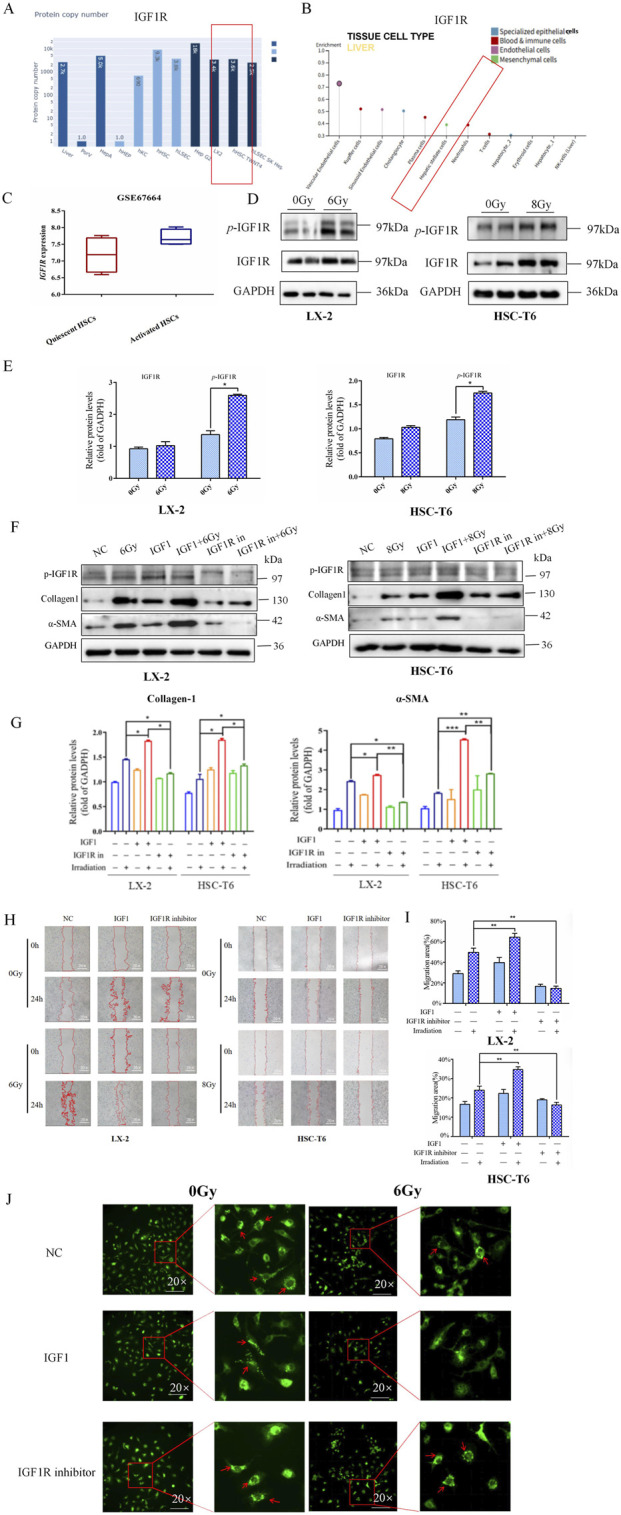
IR led to the upregulation of IGF1R, and the activation of IGF1R promoted the IR-induced HSCs activation. **(A)** Results from the Human Liver Proteome Database showed the expression of IGF1R in human liver. **(B)** Results from the Human Protein Atlas database showing the specific expression in different cells of the liver. **(C)** The comparison of the GEO database on differential expression of IGF1R in quiescent and activated HSCs. **(D)** Western blot measured the levels of *p*-IGF1R and IGF1R in LX-2 and HSC-T6 cells 3 h after 6 Gy or 8 Gy irradiation. **(E)** The quantitative analysis of the *p*-IGF1R and IGF1R levels in LX-2 and HSC-T6 cells 3 h after 6 Gy or 8 Gy irradiation. **(F)** The levels of collagen-1 and α-SMA in LX-2 and HSC-T6 cells 24 h after treatment with the IGF1R agonist or inhibitor, respectively, or after co-irradiation with 6 Gy or 8 Gy irradiation, measured using Western blot. **(G)** The quantitative analysis of the collagen-1 and α-SMA levels in LX-2 and HSC-T6 cells after treatment with the IGF1R agonist or inhibitor, respectively, or after co-irradiation with 6 Gy or 8 Gy irradiation. **(H)** The migration capability of LX-2 and HSC-T6 cells 24 h after treatment with the IGF1R agonist or inhibitor, respectively, or after co-irradiation with 6 Gy or 8 Gy irradiation, measured using the wound healing assay. **(I)** The quantitative analysis of the level of the migration capability of LX-2 and HSC-T6 cells after treatment with the IGF1R agonist or inhibitor, respectively, or after co-irradiation with 6 Gy or 8 Gy irradiation. **(J)** The intracellular lipid droplet content of LX-2 cells 24 h after treatment with the IGF1R agonist or inhibitor, respectively, or after co-irradiation with 6 Gy irradiation, measured using BODIPY staining.

Given that alterations in cellular phenotypes are governed by regulatory mechanisms of gene expression, we subsequently focused on investigating candidate genes involved in IR-induced activation of HSCs. Historically, the IGF1R family has been extensively characterized as critical mediators in HSC activation and liver fibrosis. This pathway’s relevance to hepatic fibrogenesis was corroborated by bioinformation analysis of the Human Liver Proteome Database, where IGF1R was identified as a significantly expressed protein in human liver tissue (Figure 3A). To elucidate the cellular localization of IGF1R within hepatic compartments, we interrogated the Human Protein Atlas database. The expression profiling revealed predominant IGF1R enrichment in liver non-parenchymal cells (NPCs), particularly within vascular endothelial cells, Kupffer cells, sinusoidal endothelial cells, cholangiocytes, plasma cells, and HSCs (Figure 3B). Through comprehensive analysis of gene expression profiles from HSCs in various activation states (GEO Dataset ID: GSE67664), we observed a modest elevation in *IGF1R* expression levels with activated HSCs compared to quiescent HSCs (Figure 3C). Meanwhile, the elevated levels of IGF1R and *p*-IGF1R expressions were observed during the process of HSC activation 3 h after IR (Figures 3D,E). This differential expression pattern suggested potential involvement of IGF1R-mediated signaling in modulating HSCs transition from quiescent to activated states.

**FIGURE 4:**
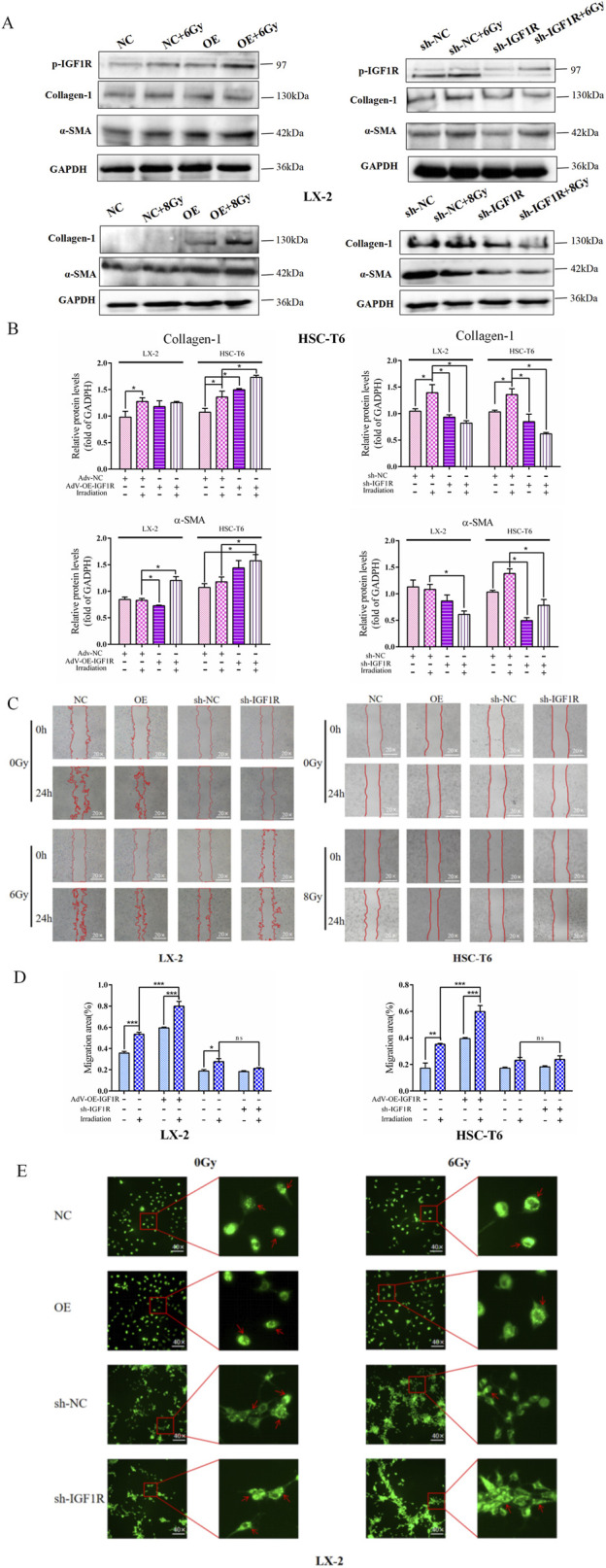
IGF1R overexpression promotes the IR-induced HSC activation. **(A)** The levels of collagen-1 and α-SMA in LX-2 and HSC-T6 cells in the OE-IGF1R and sh-IGF1R groups compared to those in the NC and sh-NC groups, respectively, or after co-irradiation 24 h following 6 Gy or 8 Gy irradiation. **(B)** The quantitative analysis of the levels of collagen-1 and α-SMA in LX-2 and HSC-T6 cells in the OE-IGF1R and sh-IGF1R groups, compared to those in the NC and sh-NC groups, respectively, or after co-irradiation with 6 Gy or 8 Gy. **(C)** The migration capability of LX-2 and HSC-T6 cells in the OE-IGF1R and sh-IGF1R groups compared to those in the NC and sh-NC groups, respectively, or after co-irradiation 24 h following 6 Gy or 8 Gy irradiation. **(D)** Quantitative analysis of the migration capability of LX-2 and HSC-T6 cells in the OE-IGF1R and sh-IGF1R groups compared to those in the NC and sh-NC groups, respectively, or after co-irradiation with 6 Gy or 8 Gy. **(E)** The intracellular lipid droplet content of LX-2 cells in the OE-IGF1R and sh-IGF1R groups compared to those in the NC and sh-NC groups, respectively, or after co-irradiation 24 h following 6 Gy irradiation.

Therefore, in a similar way, we wondered whether IGF1R has the potential to modulate IR-induced HSCs activation. In the LX-2 and HSC-T6 cell line models, both the IGF1R agonist (IGF1) and inhibitor (AZD-3463) were used with the objective of either activating or inhibiting IGF1R activity. The results demonstrated that the increased α-SMA and collagen-1 expressions (Figures 3F,G) were accompanied by a significant expansion in cell migration areas in the IGF1 pre-treatment combined with irradiation group compared to the irradiation-only group (Figures 3H,I). The intracellular lipid droplets were significantly reduced to such an extent that they were undetectable under the microscope in IGF1 combined irradiation treatment group, and the inhibitor AZD-3463 treatment effectively prevented IR induced depletion of lipid droplets in activated HSCs (Figure 3J; Supplementary Figure S1). To further determine whether IGF1R redundancy or deficiency could influence the progression of HSCs activation, we constructed IGF1R overexpression- and knockdown-stable transfected cell lines (OE and sh-IGF1R), with negative vectors as the control group (NC and sh-NC) (Supplementary Figure S2). Increased expression of indicators related to cellular activation after IR was exacerbated by IGF1R overexpression, which was significantly inhibited by IGF1R knockdown. The specific manifestations were largely consistent with the use of inhibitors and agonists such as elevated α-SMA and collagen-1 expressions (Figures 4A,B), enhanced cell migration ability (Figures 4C,D), and significant loss of lipid droplets in the OE co-irradiation group, whereas the results in the sh-IGF1R co-irradiation group were diametrically opposite (Figure 4E; Supplementary Figure S3). Thus, these results suggest that ionizing radiation can lead to an increase in IGF1R expression and has the potential to serve as a target intervention to block or inhibit the activation of HSCs.

### 3.4 IGF1R promotes IR-induced HSCs activation mediated through the DNA damage repair process activated by DNA-PKcs

**FIGURE 5:**
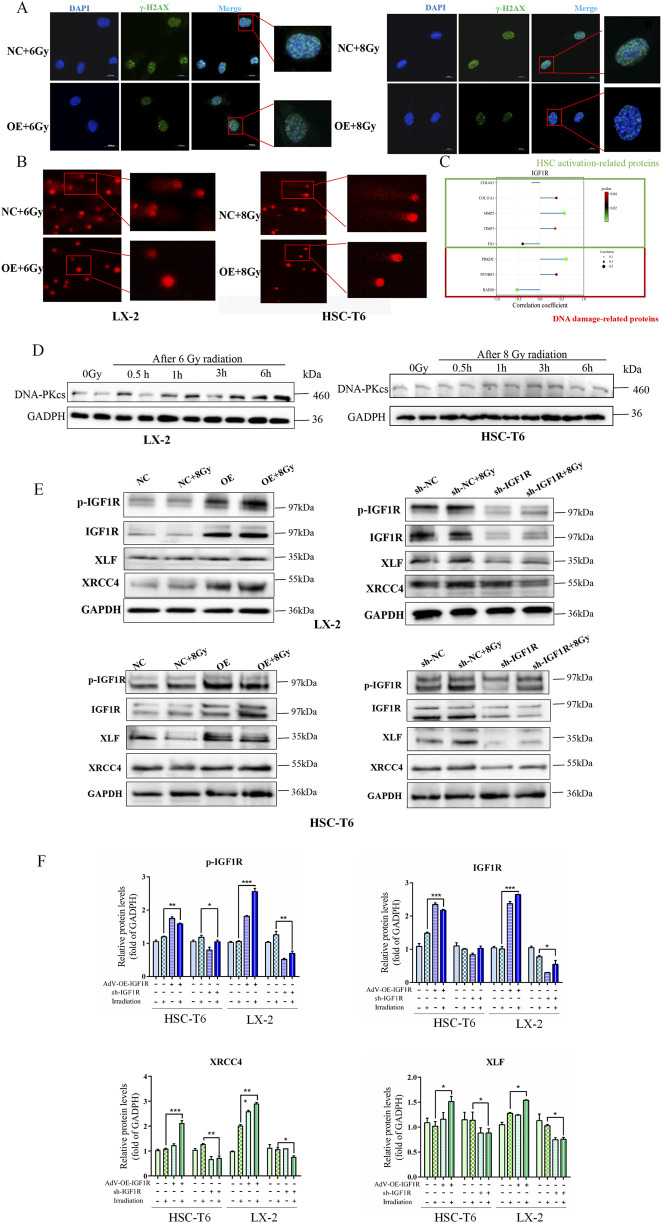
Role of IGF1R in promotion of IR-induced HSCs activation mediated through the DNA damage repair process activated by DNA-PKcs. **(A)** Level of DNA damage foci of γ-H2AX in LX-2 and HSC-T6 cells in the NC and OE groups after co-irradiation with 6 Gy or 8 Gy, detected 3 h after IR. The cell nuclei were counterstained with DAPI (blue), and γ-H2AX was stained green, measured by IF. **(B)** DNA damage in LX-2 and HSC-T6 cells, measured using the alkaline comet assay; the length of the comet tail represents the severity of DNA damage. **(C)** Results of correlation analysis of IGF1R with fibrosis-related genes and DNA damage-related genes in human liver fibrosis samples using Spearman’s correlation coefficient using data from the GEO database. **(D)** The DNA-PKcs expression level detection in the DNA damage time point of the HSC-T6 cell activation process induced by irradiation at 0.5 h, 1 h, 3 h, and 6 h after IR. **(E)** The levels of p-IGF1R, IGF1R, XRCC4, and XLF in LX-2 and HSC-T6 cells in the OE and sh-IGF1R groups compared to those in the NC and sh-NC groups, respectively, or after co-irradiation with 6 Gy or 8 Gy detected 3 h after IR. **(F)** The quantitative analysis of the levels of p-IGF1R, IGF1R, XRCC4, and XLF in LX-2 and HSC-T6 cells in the OE and sh-IGF1R groups compared to those in the NC and sh-NC groups, respectively, or after co-irradiation with 6 Gy or 8 Gy.

To determine whether the activation of HSCs by IGF1R is mediated by DNA damage repair, we first investigated the effects of IGF1R on DNA damage and repair during HSCs activation. We found that a significant decrease in the fluorescence intensity of the DNA damage foci of γ-H2AX by immunofluorescence was in parallel with the massive expression of IGF1R in the OE group (Figure 5A). DNA damage was further assessed using the alkaline comet assay, which reflects DNA strand breaks by measuring the percentage of DNA in the tail moment. The results showed that the tail moment was lower in irradiation-exposed IGF1R OE cells than that in the NC cells (Figure 5B). Furthermore, to verify the synergistic nature of the action of IGF1R with DNA-PKcs during HSC activation and the existence of interactions, we reviewed the GEO database (GEO Dataset ID: GSE25097) and found that there was a correlation between IGF1/IGF1R and the expression of DNA-PKcs, along with changes in the expression of genes related to HSCs activation (Figure 5C).

Based on previous literature reports, IGF1R may regulate DNA damage repair through the NHEJ pathway. In light of the regulatory relationship between IGF1R and DNA-PKcs identified in our analysis, we investigated whether there was a positive correlation in expression levels between DNA-PKcs and IGF1R during the DNA damage repair process. The results showed that under radiation-induced HSCs activation conditions, DNA-PKcs expression significantly increased, which was consistent with the findings regarding IGF1R (Figure 5D). Furthermore, subsequent to the administration of IR, alterations were identified in the expression levels of DNA-PKcs-associated downstream proteins, XRCC4 and XLF, while changes in α-SMA and collagen-1 levels were observed in the OE and sh-IGF1R groups compared to the related NC group (Figures 5D,E). The present study demonstrated a consistent correlation between alterations in IGF1R and the trend of changes in DNA-PKcs, α-SMA, and collagen-1. The results indicated that high expression of IGF1R coincided with the expressions of DNA-PKcs, XRCC4, XLF, α-SMA, and collagen-1, and *vice versa*.

**FIGURE 6:**
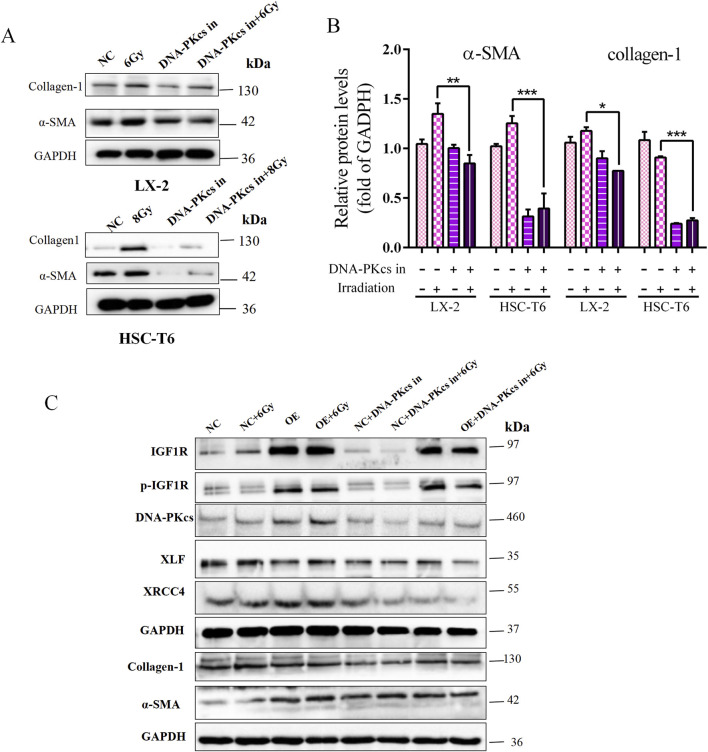
Role of IGF1R in promoting IR-induced HSCs activation, mediated through the DNA damage repair process activated by DNA-PKcs. **(A)** Levels of α-SMA and collagen-1 in LX-2 and HSC-T6 cells treated with the DNA-PKcs inhibitor, respectively, or after co-irradiation. **(B)** Quantitative analysis of the levels of α-SMA and collagen-1 in LX-2 and HSC-T6 cells treated with the DNA-PKcs inhibitor, respectively, or after co-irradiation. **(C)** The levels of p-IGF1R, IGF1R, XRCC4, XLF, α-SMA, and collagen-1 in LX-2 cells in the OE and NC groups treated with the DNA-PKcs inhibitor, respectively, or after co-irradiation with 6 Gy.

Then, HSCs were treated with the DNA-PKcs inhibitor STL127705 to inhibit DNA damage repair; we observed that α-SMA and collagen-1 expression levels were clearly downregulated (Figures 6A,B), indicating that DNA-PKcs-mediated DNA damage repair promoted radiation-induced HSCs activation.

To provide further clarification regarding the ability of IGF1R to promote HSCs activation and DNA damage repair, it is important to note that this is attributed to the differential expression of DNA-PKcs after IR treatment. In the present study, STL127705 was used in irradiated IGF1R OE cell lines and NC cells, with the objective of inhibiting the activity of DNA-PKcs. The results obtained demonstrated that, compared with the irradiated group alone, the expression of DNA damage proteins XRCC4 and XLF, which are downstream of DNA-PKcs, was significantly reduced following the administration of STL127705. Additionally, the level of HSC activation marker genes was reduced. This change was not alleviated even in the presence of IGF1R overexpression (Figure 6C). The above results indicated that the involvement of IGF1R in HSCs activation is, at least in part, mediated through the DNA damage repair process activated by DNA-PKcs, a key protein in the NHEJ repair process.

### 3.5 IGF1R exerted its DNA damage repair effects by affecting *PRKDC* transcription and nucleoplasmic transport of DNA-PKcs

**FIGURE 7:**
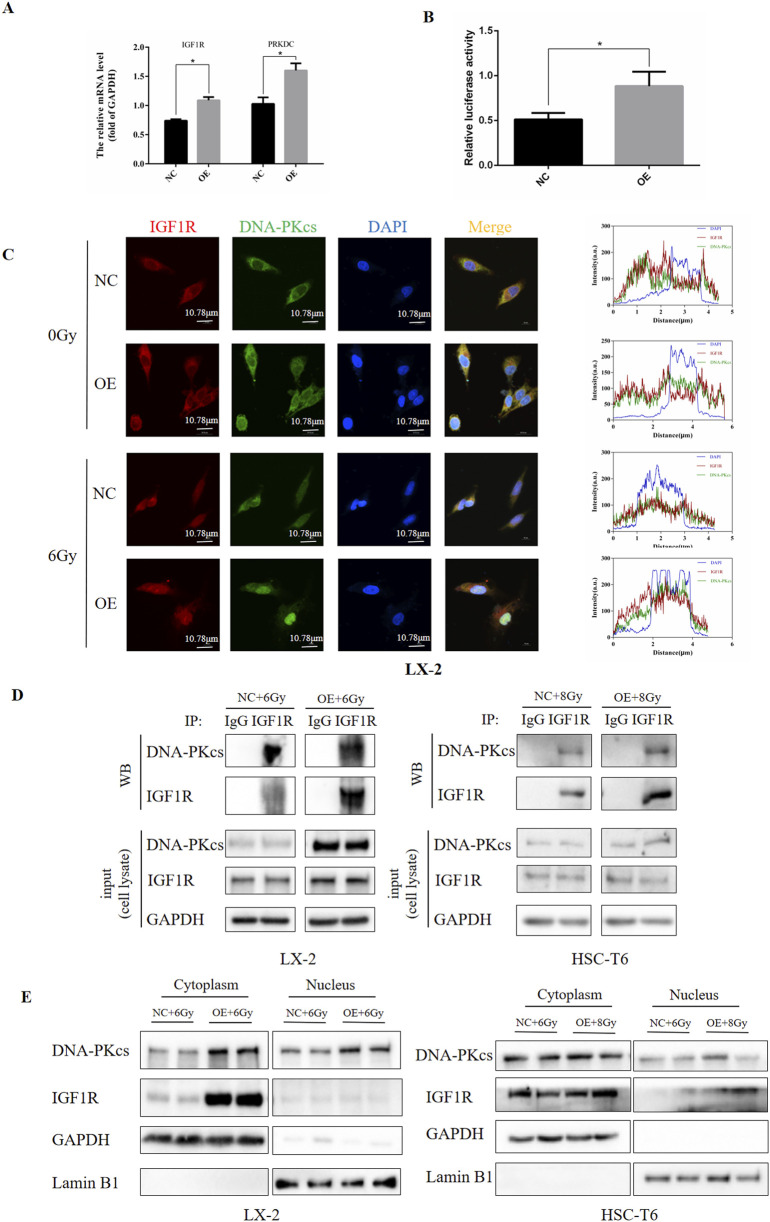
IGF1R exerted its DNA damage repair effects by affecting PRKDC transcription and nucleoplasmic transport of DNA-PKcs. **(A)** Levels of *IGF1R* and *PRKDC* affected by IGF1R in LX-2 cells, measured by RT-qPCR. **(B)** Ability of IGF1R to act as a transcription factor in the PRKDC promoter region in LX-2 cells, measured using dual-luciferase reporter assays. **(C)** Levels of IGF1R and DNA-PKcs in LX-2 and HSC-T6 cells in the NC and OE groups after co-irradiation with 6 Gy. The cell nuclei were counterstained with DAPI (blue), IGF1R was stained red and DNA-PKcs was stained green. Protein immunofluorescence co-localization analysis was performed using ZEISS ZEN 3.8 software **(D)** Levels of IGF1R and DNA-PKcs and the binding of these two proteins in LX-2 and HSC-T6 cells in the NC and OE groups after co-irradiation with 6 Gy or 8 Gy, measured using the CO-IP assay. **(E)** The expressions of IGF1R and DNA-PKcs in the nucleus and cytoplasm after nuclear–cytoplasmic fractionation in LX-2 and HSC-T6 cells in the NC and OE-IGF1R group after co-irradiation with 6 Gy or 8 Gy, measured by Western blot.

Having established that IGF1R regulates DNA-PKcs, we sought to determine whether this regulatory mechanism operates through transcriptional control of *PRKDC* at the RNA level or via modulation of DNA-PKcs protein activity. To investigate transcriptional regulation, we performed dual-luciferase reporter assays and q-PCR in LX-2 cells, evaluating the potential of IGF1R as a PRKDC transcription factor. Notably, in irradiated LX-2 cells overexpressing IGF1R, we observed a significant increase in firefly *PRKDC* expression luciferase activity compared to that in irradiation-only controls (Figure 7A). This demonstrates that IGF1R enhances PRKDC promoter activity, suggesting its role as a transcriptional regulator of DNA-PKcs expression.

To complement our transcriptional findings, we examined the functional relationship between IGF1R and DNA-PKcs at the protein level. Immunofluorescence analysis in HSCs revealed robust cytoplasmic and nuclear co-localization of IGF1R and DNA-PKcs, suggesting potential physical interaction. Strikingly, IGF1R-overexpressing cells subjected to IR exhibited an increase in nuclear DNA-PKcs fluorescence intensity compared to irradiated controls (Figure 7B). Although nuclear localization of DNA-PKcs aligns with its canonical role in DNA damage repair, the concurrent nuclear detection of IGF1R, which was a receptor tyrosine kinase traditionally associated with membrane signaling, represents a novel observation. This rewarding spatial redistribution implies a non-canonical mechanism by which IGF1R may directly regulate DNA-PKcs.

Based on these findings, we first searched for IGF1R binding proteins using an anti-IGF1R antibody through the co-IP assay. This revealed a constitutive physical interaction between endogenous IGF1R and DNA-PKcs, which was significantly enhanced in IGF1R OE cells (Figure 7C). Subsequently, by separating the nuclear and cytoplasmic proteins of subcellular fractionation experiments, we found that IGF1R OE cells exhibited an increase in nuclear DNA-PKcs accumulation compared to irradiated controls, concurrent with a marked cytoplasmic enrichment of IGF1R. Notably, low but detectable levels of IGF1R were observed in nuclear fractions (Figure 7D; Supplementary Figure S4).

Collectively, these results suggest that IGF1R mediates IR-induced HSC DNA damage repair by regulating *PRKDC* transcription and DNA-PKcs activity at both the RNA and protein levels.

## 4 Discussion

IR-induced liver fibrosis is an unavoidable complication of radiotherapy for liver cancer and abdominal malignancies, posing a significant socioeconomic burden on patients and healthcare systems worldwide (Jiang et al., 2023; Stauffer et al., 2024). The core of this radiobiological phenomenon lies in the activation of radiation-sensitive HSCs, which drives liver fibrosis through dynamic cellular reprogramming (Higashi et al., 2017; Sun et al., 2024; Poreba and Durzynska, 2020). Fortunately, emerging evidence suggests the potential reversibility of liver fibrosis through targeted intervention during the early HSC activation stage, thereby preventing the progression to irreversible cirrhosis and even hepatocellular carcinoma (Yang et al., 2020; Yang C. et al., 2021; Lee et al., 2024; Moon et al., 2020). Although substantial advancements have been made in elucidating the molecular mechanisms underlying fibrotic pathogenesis and regression, critical gaps persist in our understanding of radiation-specific activation pathways.

First but not least, this study provided the first systematic elucidation of the temporal dynamics of HSC activation following IR exposure. We established definitive dose–response relationships and temporal patterns governing both HSC activation and DNA damage in irradiated HSCs. The methodology framework developed herein offered a robust paradigm for characterizing radiation-specific HSCs cellular responses, serving as a reference for future investigations into hepatic radiobiology and therapeutic countermeasure development.

Our investigations revealed that radiation-induced HSC activation shares fundamental phenotypic characteristics with TGF-β-mediated activation, including cytoskeletal remodeling, enhanced migratory capacity, lipid droplet depletion, and ECM deposition (Kamm and McCommis, 2022; Yang F. et al., 2021). Notably, we identified a critical distinction in irradiation-exposed HSC activation, which exhibited persistent DNA damage response signatures accompanied by DNA damage repair via the NHEJ mechanism. These observations collectively support the hypothesis that strategic modulation of radiation-induced DNA damage signaling during the initial activation phase may disrupt HSCs transdifferentiation and consequently mitigate fibrotic progression.

The presence of DNA damage-related response and repair defects after HSCs activation is a hot topic of current research. Therefore, targeting the DNA damage repair pathway is of high priority as an antifibrotic strategy (Arroyo et al., 2021; Zhang et al., 2022; Meng et al., 2018; Gan et al., 2025). Currently, most of the studies on DNA damage repair focus on the repair of single- and double-stranded DNA breaks, and there are two main repair modes of DNA strand breaks, namely, NHEJ and HR, of which NHEJ repair is the most common and accounts for approximately 70% of the total (Cheng et al., 2020; Parola and Pinzani, 2019; Iredale et al., 2013). DNA-PKcs is a key molecule in cellular DSB NHEJ repair, and its kinase activity determines its repair function for DSBs; the expression of DNA-PKcs is associated with liver fibrosis (Bai et al., 2024). Our results demonstrated that targeted inhibition of DNA-PKcs after IR effectively reduces the expression of HSC activation marker genes.

Many studies indicate that IGF1R can promote the proliferation and transformation of HSCs into myofibroblast-like cells through activation of classical pathways, such as PI3K/AKT and MAPK/ERK, which, in turn, secrete a large amount of ECM and are closely associated with hepatic fibrosis (Riedemann and Macaulay, 2006; Zou et al., 2022). To verify that IGF1R can also play a pro-fibrotic role during IR-induced HSC activation, we initially elucidated its biological function by affecting IGF1R activity and intracellular abundance through IGF1R inhibitors and agonists or by overexpressing or interfering with IGF1R expression, and we observed that the activation phenotypes of HSCs were altered accordingly, including cell migration and cellular lipid droplets, suggesting the critical role of IGF1R in IR-induced HSCs activation, which should not be underestimated.

What is more noteworthy is that in recent years, it has been reported that IGF1R can participate in the radiation-induced DNA damage repair pathway (Wu et al., 2022; Wei et al., 2024), but how it intervenes in this process has rarely been reported. Meanwhile, some studies have found that IGF1R, as a membrane receptor protein, can translocate to the nucleus and that the nucleus-localized nIGF1R can interact with DNA-PKcs (Chrudinová et al., 2024). However, whether it regulates IR-induced HSCs activation through DNA-PKcs-mediated DNA damage repair is not known. Our study demonstrated that DNA-PKcs exhibited expression changes with IGF1R in IR-induced HSCs activation, and IGF1R expression was negatively correlated with DNA damage levels. This effect was mediated by XRCC4 and XLF, the downstream proteins of DNK-PKcs in the NHEJ-related DNA damage repair process. When we addressed the HSC activation and DNA damage repair in DNA-PKcs inhibitor-treated IGF1R-overexpressed cells, the results showed that the promotion of IGF1R in IR-induced HSCs activation was alleviated by the inhibition of DNA-PKcs and its roles in DNA damage repair, indicating the role of IGF1R in the IR-induced HSCs activation process mediated by DNA-PKcs. The next issue to clarify is how IGF1R mediates DNA damage repair through DNA-PKcs in this process.

The interaction between IGF1R and DNA-PKcs is extremely high in both the nucleus and cytoplasm, at the same time, when IGF1R is highly expressed, DNA-PKcs is also highly expressed. Under IR treatment, the binding level of IGF1R with DNA-PKcs was higher in the nucleus, suggesting that IGF1R may promote DNA damage repair by increasing DNA-PKcs expression and facilitating its nuclear translocation through interaction with DNA-PKcs.

In conclusion, this study confirmed that IGF1R promotes the activation of HSCs by enhancing DNA damage repair through the DNA-PKcs-mediated NHEJ pathway; the mechanism was preliminarily clarified as IGF1R upregulating the expression of DNA-PKcs and facilitating its nuclear translocation through interaction with DNA-PKcs. However, a limitation of this study is that it focused only on the relationship between DNA damage caused by ionizing radiation and the activation of HSCs at the cellular level, without further studies in animal models.

## Data Availability

The datasets presented in this study can be found in online repositories. The names of the repository/repositories and accession number(s) can be found in the article/Supplementary Material.
