# Supplementary material for: IGF1R promotes radiation-induced HSCs activation by regulating DNA-PKcs-mediated DNA damage repair
Source: Front Cell Dev Biol. 2025 Nov 14;13:1678654. doi: 10.3389/fcell.2025.1678654 (PMC12660224; doi:10.3389/fcell.2025.1678654)
Supplement: Supplementary file 1 [file DataSheet1.docx]

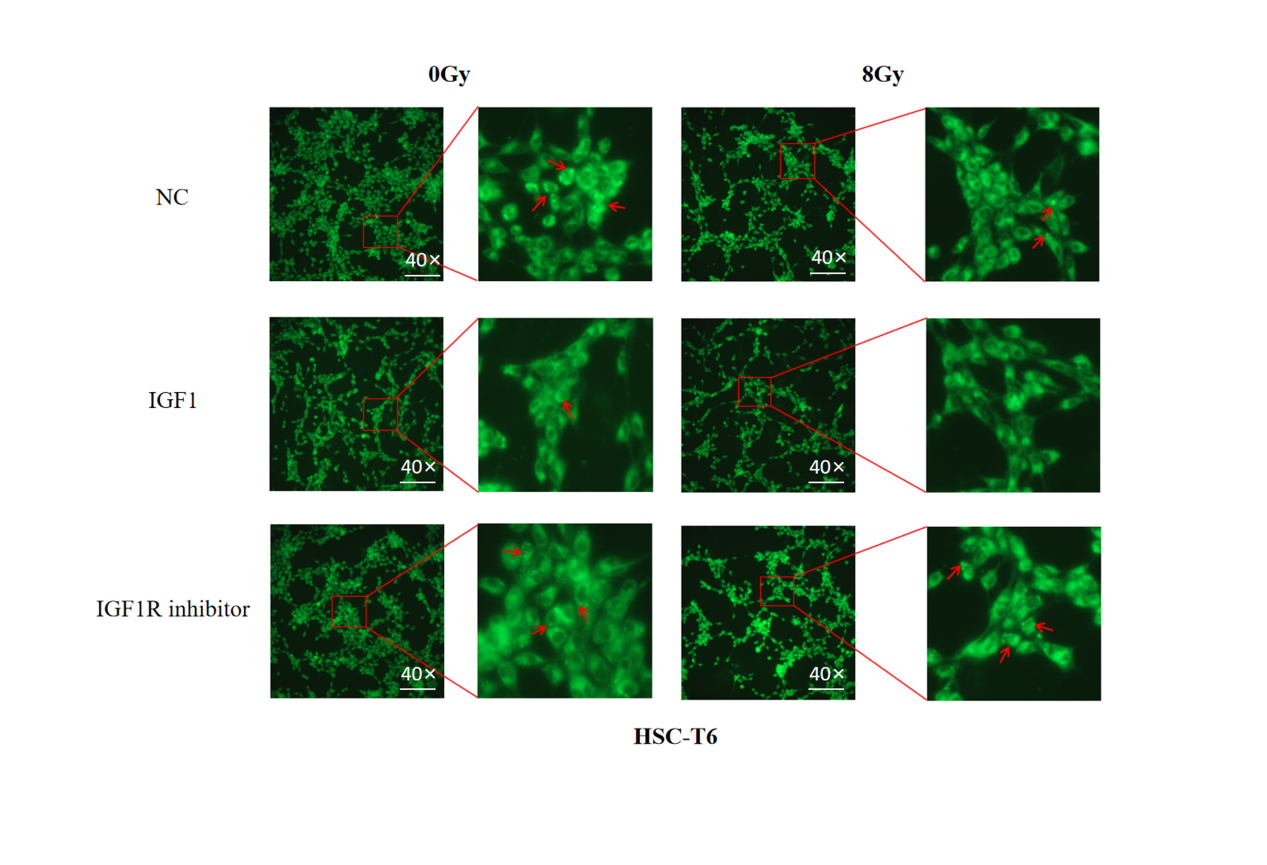


**Figure S1** **IR led to upregulation of IGF1R and IGF1R promote the IR induced HSCs activation.** BODIPY staining measured the intracellular lipid droplet content of HSC-T6 cells after treatment of IGF1R agonist or inhibitor respectively or co-irradiation of 8Gy.


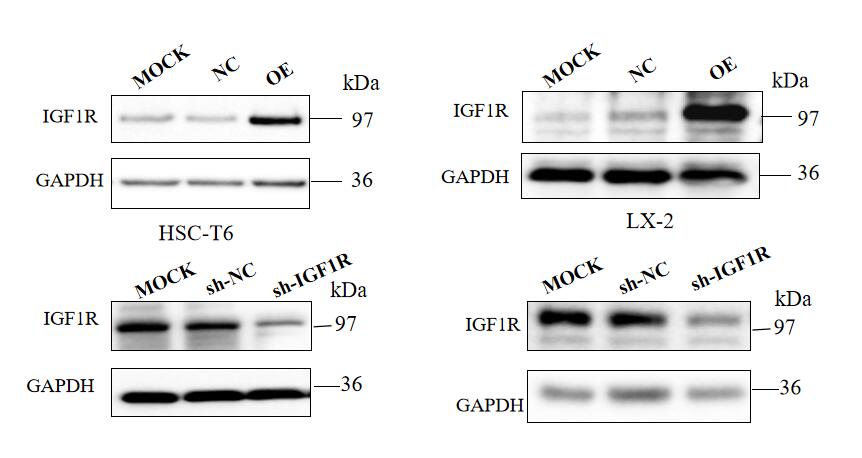


**Figure S2**  **IR led to the upregulation of IGF1R and IGF1R promote the IR induced HSCs activation.** The level of IGF1R in LX-2 and HSC-T6 cells at OE and sh-IGF1R groups compared to NC and sh-NC groups respectively.


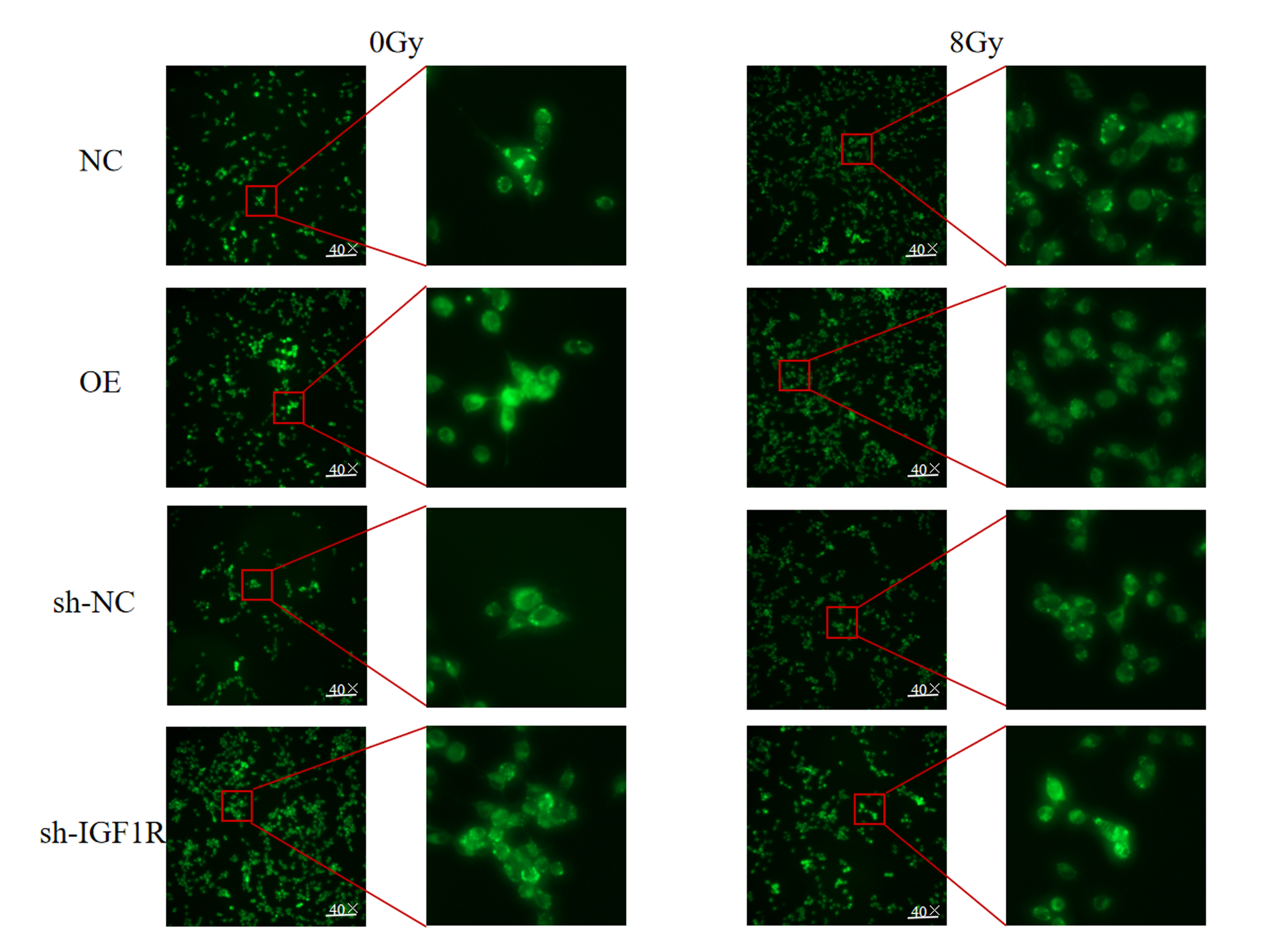


**Figure S3** **IGF1R overexpression promote the IR induced HSCs activation.** The intracellular lipid droplet content of HSC-T6 cells in OE and sh-IGF1R groups compared to NC and sh-NC groups respectively or co-irradiation of 8Gy.


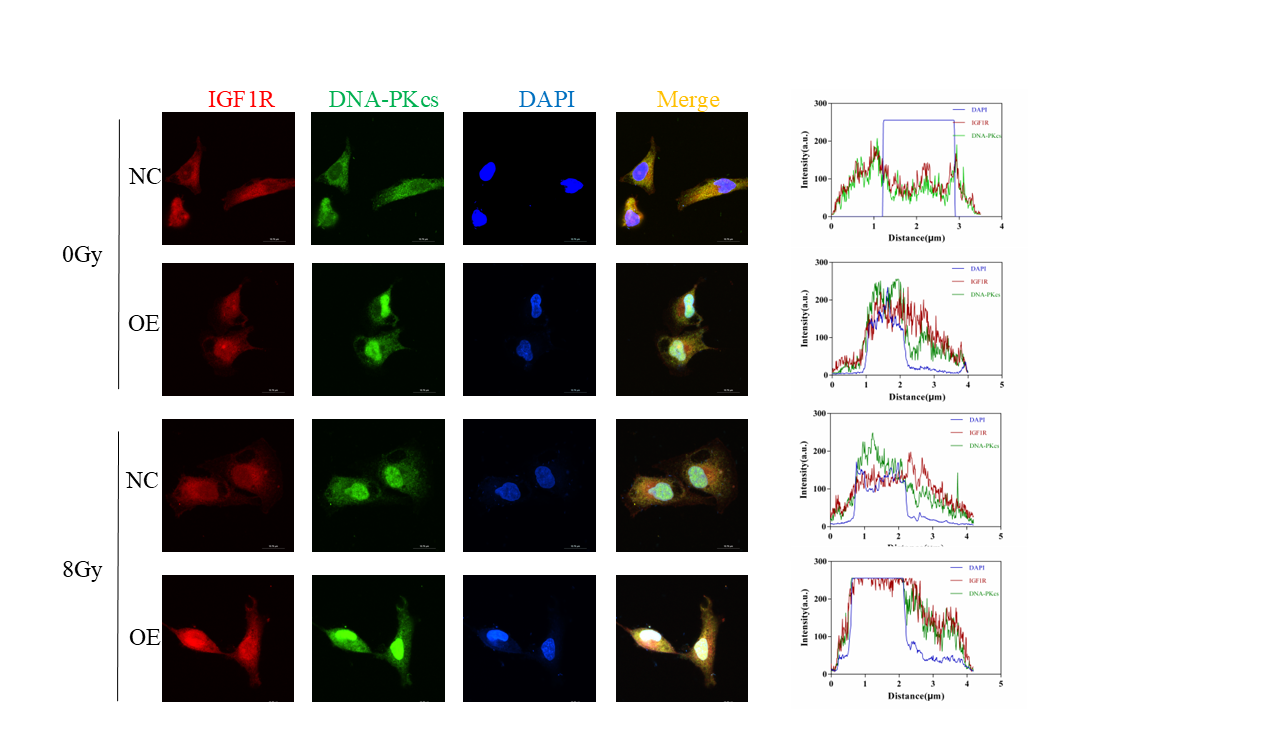


**Figure S4** **IGF1R exerted its DNA damage repair effects by affecting *PRKDC* transcription and nucleoplasmic transport of DNA-PKcs.** The level of IGF1R and DNA-PKcs in HSC-T6 cells at NC and OE groups co-irradiation of 6Gy, cell nuclei were counterstained with DAPI (blue), IGF1R were stained with red and DNA-PKcs were stained with green. The protein immunofluorescence co-localization analysis presented by ZEISS ZEN 3.8 software.
